# Mitochondrial calcium uniporter is required for thermogenic adaptation mediated by reactive oxygen species signaling

**DOI:** 10.1016/j.jlr.2025.100834

**Published:** 2025-05-29

**Authors:** Suji Kim, Seung-Kuy Cha, Kyu-Sang Park, Jun Namkung

**Affiliations:** 1 Organelle Medicine Research Center, Yonsei University Wonju College of Medicine, Wonju, Republic of Korea; 2 Department of Biochemistry, Yonsei University Wonju College of Medicine, Wonju, Republic of Korea; 3 Department of Physiology, Yonsei University Wonju College of Medicine, Wonju, Republic of Korea; 4 Department of Global Medical Science, Yonsei University Wonju College of Medicine, Wonju, Republic of Korea

**Keywords:** mitochondria, adipose tissue, brown, dipocytes, besity, lipolysis and fatty acid metabolism

## Abstract

Mitochondrial Ca^2+^ influx via mitochondrial calcium uniporter (MCU) accelerates mitochondrial biogenesis and energy metabolism. Nevertheless, the molecular mechanism of MCU-dependent mitochondrial activation and thermogenesis in thermogenic adipose tissues remains elusive. In this study, we demonstrate that MCU governs mitochondrial functions in brown and beige adipocytes via the formation of mitochondrial reactive oxygen species (mtROS). Mice with a brown adipose tissue-specific *Mcu* knockout (*Mcu* BKO) mice exhibited decreased oxygen consumption and heat production, accompanied by downregulation of genes related to β-oxidation and thermogenesis. Furthermore, *Mcu* BKO mice, exhibiting a reduction in mtROS, showed defective thermogenic responses to cold exposure or β-adrenergic stimulation. Downregulation of thermogenic genes including *Ucp1* in *Mcu* BKO mice can be rescued by exogenous ROS through AMP-activated protein kinase (AMPK) activation. Collectively, our findings suggest that MCU modulates mtROS-mediated mitonuclear signaling in thermogenic adipocytes.

## Graphical Abstract

**Figure:**
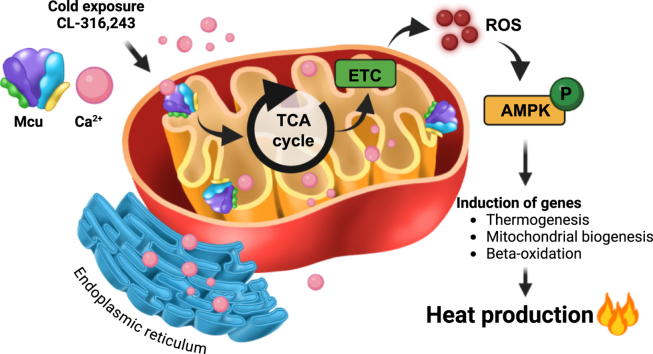

Thermogenic adipocytes, particularly brown and beige adipocytes, contribute to systemic energy metabolism by dissipating energy as heat through both UCP1-dependent and UCP1-independent mechanisms, contributing to non-shivering thermogenesis (NST) (1, 2, 3). These specialized cells are distinguished from energy-storing white adipocytes by a high number of mitochondria and unique metabolic abilities, including uncoupling of mitochondrial electron transport from ATP production, which facilitates thermogenesis and energy expenditure (4). Mitochondrial activity can be modulated by the influx of calcium ions (Ca^2+^) into the mitochondria. Ca^2+^ released from endoplasmic reticulum (ER) through inositol-1,4,5-triphosphate (IP_3_) receptor is transported into the mitochondrial matrix via the voltage-dependent anion channel (VDAC) and the mitochondrial calcium uniporter (MCU) complex, located on the outer and inner mitochondrial membrane, respectively (5, 6). The MCU complex comprises the pore-forming MCU and its regulators, including MCU dominant-negative β-subunit (MCUb), essential MCU regulator (EMRE), and mitochondrial calcium uptake (MICU) 1 and 2 (7, 8). The influx of calcium into the mitochondria is known to enhance the activity of proteins responsible for the citric acid cycle and electron transfer, thereby activating mitochondrial bioenergetics (5, 9). Thermogenic function of cellular Ca^2+^ is known to be involved in the Ca^2+^ futile cycle at the ER, which is UCP1-independent noncanonical thermogenesis powered by ATP-utilizing SERCA2b (10). In response to norepinephrine during cold exposure, activation of adrenergic receptors—including α1 and β subtypes (β1, β2, and β3)—increase not only the flux of Ca^2+^ cycling but also cytosolic Ca^2+^. Although increased cytosolic Ca^2+^ entering the mitochondria can be expected to activate the process of ATP synthesis for further ER Ca^2+^ cycling, the amount and activity of ATP synthase are low in the mitochondria of brown adipose tissue due to the uncoupling of electrochemical gradient and ATP synthesis (11, 12). This leads us to hypothesize that mitochondrial Ca^2+^ mediated by MCU may have distinct functions other than ATP supply for thermogenic processes, including Ca^2+^ futile cycling, creatine futile cycling, and TAG-fatty acid cycling (13). Therefore, we initiated this research to explore the specific functions of MCU in thermogenic adipocytes. In this study, we generate brown adipocyte-specific *Mcu* knockout (*Mcu* BKO) mice and investigate their mitochondrial bioenergetics. The absence of *Mcu* resulted in decreased oxygen consumption and β-oxidation with cold intolerance. We found diminished reactive oxygen species (ROS) production and activation of AMP-activated protein kinase (AMPK) in *Mcu* BKO mice are essential mitonuclear signals for activation of thermogenic adipocytes.

## Materials and Methods

### Materials

Key reagents or resources are listed in supplemental Table S1.

### Mice

All animal protocols have been approved by the Yonsei University Wonju College of Medicine Institutional Animal Care and Use Committee (approval number: YWC-181022-1). To generate brown adipocyte-specific *Mcu* KO mice, *Mcu* floxed mice (The Jackson Laboratory, 029817) were crossed with *Ucp1* promoter-driven *Cre*-expressing mice (The Jackson Laboratory, 024670). All mice used in this study were maintained on a genetic background of *C57BL/6J* achieved through over 10 generations of backcrossing. The mice were housed in a specific pathogen-free barrier facility with a controlled climate under a 12-h light–dark cycle and provided diet and water ad libitum.

### Indirect calorimetry and body composition measurements

The metabolic ratio of mice was assessed using a Comprehensive Lab Animal Monitoring System (Columbus Instrument). Mice were individually placed in metabolic chambers, and oxygen consumption and carbon dioxide production were continuously monitored for a period of 2–3 days. During the measurement, mice had free access to food and water. Fat mass and lean mass were measured using nuclear magnetic resonance (Bruker, LF50).

### Temperature monitoring

A week before conducting fasting-cold experiments, temperature probes were implanted. Briefly, mice were anesthetized, and a telemetric sensor (STARR, G2 E-Mitter) was an abdominal cavity implanted. The body temperature was measured at 10-min intervals. For the non-invasive temperature measurement, thermal imaging camera (FLIR T530) was employed. Each group was subjected to experiments in a controlled laboratory environment at both room temperature (24°C) and cold temperature (4°C). The collected temperature data were analyzed for reproducibility by conducting measurements three times.

### Glucose tolerance test and insulin tolerance test

Glucose tolerance test (GTT) was conducted at 16 weeks of age. Following an overnight fast and a baseline blood glucose measurement, a glucose solution (2 mg/kg body weight) was administered via intraperitoneal injection. Blood glucose levels were monitored at specified time intervals (0–120 min). The insulin tolerance test (ITT) was conducted at 17 weeks of age. After 6 h fast, baseline blood glucose levels were measured. Insulin (0.45 U/kg body weight) was administered intraperitoneally, and blood glucose levels were measured at 0–60 min post-injection. The area under the curve (AUC) was calculated to evaluate glucose tolerance and insulin tolerance.

### Hematoxylin and eosin staining

Adipose tissues were fixed with 10% formalin and subsequently embedded in paraffin. Tissue sections were sliced at a thickness of 8 μm and mounted on slides. Following deparaffinization, rehydration, and hematoxylin and eosin (H&E) staining procedures carried out according to standard protocols, the stained slides were examined under an optical microscope for structural analysis.

### Real-time PCR

Total RNA was extracted using Trizol reagent (Invitrogen). The extracted RNA was further purified using an RNA purification kit (Thermo Fisher Scientific). cDNA was synthesized using a reverse transcription kit (Thermo Fisher Scientific). Quantitative PCR was performed using the Quant-Studio 6 Real-Time PCR System (Thermo Fisher Scientific). The expression levels of target mRNAs were normalized to the expressions of *Rplp0*. The sequences of primers used in this study are listed in the Supplementary Materials.

### Western blotting

For protein extraction, samples were homogenized in an ice-cold RIPA lysis buffer supplemented with protease and phosphatase inhibitors (Thermo Fisher Scientific). The total protein concentration in the lysates was determined using the BCA Protein Assay Kit (Thermo Fisher Scientific). Total cellular protein (1–20 μg) was separated on SDS-PAGE gels and transferred onto PVDF membranes using a wet transfer system according to the manufacturer's guidelines. The PVDF membranes were blocked in 5% skim milk for a 1 h to prevent nonspecific binding. Subsequently, the membranes were incubated with primary antibodies against the target proteins overnight at 4°C. Protein bands were visualized using an enhanced chemiluminescence (ECL) reagent (Cytiva Amersham) and imaged with a BIO-RAD ChemiDoc Imaging System. Quantitative data were expressed after normalized by the loading control, β-actin.

### Primary adipocyte culture

For primary brown and white cells, brown adipose tissue (BAT) and inguinal white adipose tissue (iWAT) were finely minced and subjected to enzymatic digestion in 5 ml of isolation buffer containing 50,000 units of collagenase D, with intermittent vortexing every 10 minutes for a total of 30 min at 37°C. Following digestion, 5 ml of DMEM/F12 medium was added to the mixture, which was then filtered using a 70 μm mesh filter. The resulting suspension was centrifuged at 200 *g* for 10 min, and the supernatant was carefully removed. The cell pellet was resuspended in 10 ml of RBC lysis buffer for 5 min to lyse red blood cells. After lysis, 15 ml of DMEM/F12 medium with 10% FBS was added, and the suspension was filtered through a 40 μm mesh filter. The filtered suspension was centrifuged again at 200 *g* for 5 min. The isolated primary cells were then resuspended and cultured in DMEM/F12 medium supplemented with 10% FBS. The following day, the growth medium was refreshed. Cells were maintained in DMEM/F12 supplemented with 10% fetal bovine serum and 1% antibiotic-antimycotic solution at 37°C in a humidified incubator with 5% CO_2_. To initiate adipogenesis, the confluent cells were treated with the first differentiation medium containing DMEM/F12, 10% FBS, 10 μg/ml insulin, 5 mM dexamethasone, and 0.5 mM isobutylmethylxanthine. After 72 h, this medium was replaced with the second differentiation medium comprising DMEM/F12, 10% FBS, and 10 μg/ml insulin. To induce browning of white adipocytes or differentiation of brown adipocytes, 2 nM T3 and 250 nM indomethacin were added. To activate brown adipocytes, cells were exposed to 1–2 μM of CL-316243 for 4–24 h.

### Oxygen consumption rate measurement

Primary brown adipocytes were subjected to metabolic profiling using the Agilent Seahorse XF Analyzer in a 96-well plate format. The cells were cultured and differentiated according to the previously mentioned protocols. On the day of the assay, the culture medium was replaced with Seahorse XF Assay Medium. Oligomycin, FCCP (Carbonyl cyanide 4-(trifluoromethoxy)phenylhydrazone), and Rotenone/Antimycin A were sequentially injected. Proton leak was calculated as relative change of ooxygen consumption rate (OCR) after oligomycin. For fatty acid oxidation, OCR at basal and after FCCP-treated cells with or without etomoxir pretreatment were measured.

### Measurement of mitochondrial and cytosolic ROS

Mitochondrial and cytosolic reactive oxygen species (ROS) levels were assessed using MitoSOX Red and 5-(and-6)-chloromethyl-2′,7′-dichlorodihydrofluorescein diacetate, acetyl ester (DSCDA) probes, respectively. Primary brown adipocytes were treated with the respective probes according to the manufacturer's instructions (Thermo Fisher Scientific) and incubated for a specified duration. Following treatment, cells were washed, and fluorescence was measured using a microplate reader for quantification.

### Measurement of mitochondria membrane potential

The cells were cultured until they reached the desired confluency. The culture medium was then removed, and the cells were washed twice with KRB buffer. JC-1 (Invitrogen) dye was prepared at a final concentration of 1 μM in KRB buffer and added to the cells. The cells were incubated with the JC-1 staining solution at 37°C for 30 min. Following incubation, the staining solution was removed, and the cells were washed with KRB buffer. KRB buffer was added to the cells before fluorescence measurements were taken. The fluorescence intensity was measured using a fluorescence microscope or plate reader, with excitation/emission wavelengths set to 490/530 nm for the monomeric form (green) and 540/590 nm for the aggregate form (red). The mitochondrial membrane potential was assessed by calculating the ratio of red to green fluorescence intensity.

### Mitochondria isolation

To isolate mitochondria, cells were cultured to the desired confluency and harvested by trypsinization, followed by centrifugation at 600*g* for 5 min at 4°C. The cell pellet was resuspended in ice-cold isolation buffer (250 mM sucrose, 1 mM EDTA, 10 mM HEPES, pH 7.4) and homogenized on ice. The homogenate was centrifuged at 700*g* for 10 min at 4°C to remove nuclei and cell debris. The supernatant was carefully transferred to a new tube and centrifuged again at 10,000 *g* for 15 min at 4°C to pellet the mitochondria. The mitochondrial pellet was washed with an ice-cold isolation buffer, and it was resuspended in a small volume of ice-cold isolation buffer for downstream applications.

### Mitochondria calcium uptake assay

The cells were initially grown until they achieved the desired confluency. After removing the culture medium, the cells were rinsed twice with calcium free KRBB buffer. A staining solution containing Calcium Green-5N (Invitrogen) dye at a final concentration of 100 nM was prepared in calcium free KRBB buffer and applied to the cells. The cells were then incubated at 37°C for 30 min to allow for adequate staining. After incubation, the staining solution was removed, cells were washed twice with calcium free KRBB. Fluorescence intensity was measured using a fluorescence microscope or a plate reader, with the excitation and emission wavelengths set at 490/516 nm. The uptake of calcium by mitochondria was determined by observing the variations in fluorescence intensity.

### Fluorescence staining

The cells were fixed with 4% paraformaldehyde for 15 min at room temperature and then washed with PBS. For mitochondrial staining, the cells were incubated with Mitotracker (Invitrogen) dye at a final concentration of 100 nM in PBS for 1 min at 37°C, followed by three washes with PBS. Next, Alexa 488 (Invitrogen) was then added at a final concentration of 400 nM and incubated for 30 min at room temperature, followed by three washes with PBS. Nuclei were stained with DAPI (Invitrogen) at a final concentration of 1 μg/ml for 1 min, followed by three washes with PBS. The stained cells were then mounted using a mounting medium. Fluorescence images were captured using a fluorescence microscope with appropriate filters for DAPI (excitation/emission: 358/461 nm), Mitotracker (excitation/emission: 579/599 nm), and Alexa 488 (excitation/emission: 495/519 nm).

### Glucose uptake assay

Glucose uptake was measured using the 2-NBDG Glucose Uptake Assay Kit (ab235976, Abcam) according to the manufacturer’s instructions. Briefly, differentiated brown adipocytes were washed and incubated with glucose-free culture medium containing 2-NBDG at a final concentration of 100 μg/ml for 2 h at 37°C. After incubation, the cells were washed and processed according to the assay protocol. Fluorescence was measured using a microplate reader (Ex/Em = 485/535 nm).

### Statistical analysis

All experimental data were presented as the mean ± standard error of the mean (SEM). Statistical analyses were performed using GraphPad Prism software, version 10. For comparisons, an unpaired two-tailed Student's *t* test or one-way analysis of variance (ANOVA) were employed. ∗*P* < 0.05, ∗∗*P* < 0.01, ∗∗∗*P* < 0.001, ∗∗∗∗*P* < 0.0001.

## Results

### MCU is required for differentiation of brown adipocytes

**Fig. 1:**
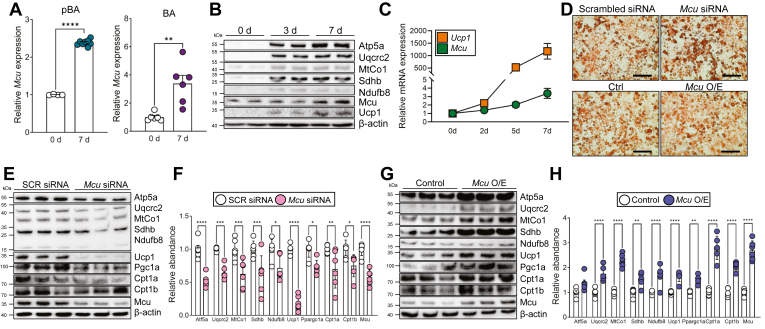
MCU is required for differentiation of brown adipocytes. A: Time-course of *Mcu* mRNA expression during the differentiation of primary and immortalized brown adipocytes. B: Protein expression of thermogenic and mitochondrial markers during differentiation. C: Temporal expression of *Ucp1* and *Mcu* mRNA during brown adipocyte differentiation. D: Oil Red O staining of brown adipocytes transfected with control, *Mcu* siRNA, or overexpression constructs. Scale bar, 400 μm. E, F: Western blot analysis of mitochondrial respiratory complex proteins upon *Mcu* knockdown. G, H: Western blot analysis of mitochondrial proteins upon *Mcu* overexpression. Data represent mean ± SEM. Statistical significance was determined using unpaired two-tailed *t* test. ∗*P* < 0.05; ∗∗*P* < 0.01; ∗∗∗*P* < 0.001; ∗∗∗∗*P* < 0.0001.

First, we examined the changes in Mcu expression during the differentiation of brown adipocytes. In both the primary brown adipocyte and its immortalized cell line, we observed a time-dependent increase in *Mcu* expression throughout the differentiation period (Fig. 1A). Concurrently, alongside other mitochondrial proteins, the protein level of Mcu also increased during this differentiation process (Fig. 1B). While it is expected for mitochondrial content to increase during differentiation (14), we observed a correlation between the expression of Ucp1 protein and mRNA with Mcu expression (Fig. 1B, C). This led us to hypothesize that Mcu expression might have a regulatory role in brown adipocyte differentiation. The lipid content of differentiated brown adipocytes was not significantly altered by overexpression or knockdown of *Mcu* (Fig. 1D). However, we detected noticeable changes in the expression of mitochondrial proteins, including electron transfer chain (ETC) components and Ucp1, which were upregulated with *Mcu* overexpression and reduced with *Mcu* knockdown (Fig. 1E–H and supplemental Fig. S1A, B). These findings strongly suggest a crucial role for Mcu in brown adipocyte differentiation.

### BAT-specific *Mcu* KO shows increased adiposity with decreased thermogenic gene expression

**Fig. 2:**
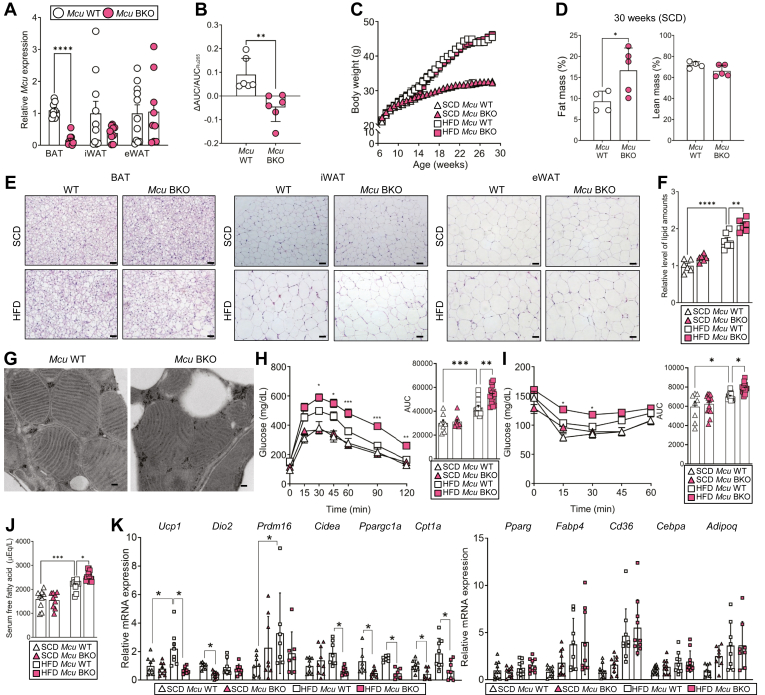
BAT-specific *Mcu* knockout leads to increased adiposity and reduced thermogenic gene expression. A: Depot-specific expression of *Mcu* mRNA in wild-type and *Mcu* BKO mice. B: Measurement of mitochondrial Ca^2+^ uptake. C: Body weight changes under standard chow diet (SCD) and high-fat diet (HFD). D: Body composition analysis of SCD-fed mice. E: Representative H&E staining of adipose depots. Scale bar, 80 μm. F: Quantification of lipid accumulation in BAT. G: Transmission electron microscopy (TEM) images of BAT. Scale bar, 100 nm. H, I: Glucose tolerance (H) and insulin tolerance (I) tests. J: Serum fatty acid concentrations. K: Expression profiles of thermogenic and lipid metabolism-related genes in BAT. Data represent mean ± SEM. Statistical significance was determined using unpaired two-tailed *t* test or one-way ANOVA. ∗*P* < 0.05; ∗∗*P* < 0.01; ∗∗∗*P* < 0.001; ∗∗∗∗*P* < 0.0001.

To further explore the functions of Mcu in brown adipose tissue, we generated brown adipocyte-specific *Mcu* knockout (*Mcu* BKO) mice using the Cre-loxP system driven by the *Ucp1* promoter (Fig. 2A). We confirmed the functional knockout of *Mcu* by diminished Ca^2+^ uptake in isolated mitochondria (Fig. 2B and supplemental Fig. S2A) and increased phosphorylation of pyruvate dehydrogenase (PDH), indicating a decline in its activity due to reduced Ca^2+^ level in mitochondrial matrix (15, 16) (supplemental Fig. S2B). Additionally, *Mcu* BKO showed decreased mitochondrial membrane potential (supplemental Fig. S2C), implying decreased flux of electron transfer due to decreased Ca^2+^ uptake. The *Mcu* BKO mice exhibited a body weight comparable to that of controls, under both standard chow diet (SCD) and high-fat diet (HFD) (Fig. 2C). However, *Mcu* BKO mice displayed increased fat mass (Fig. 2D) and higher lipid contents in their adipose tissues (Fig. 2E, F and supplemental Fig. S2D). Electron microscopy analysis revealed that *Mcu* BKO mice had larger lipid droplets, although their mitochondrial structures and contents were similar to those of wild type mice (Fig. 2G and supplemental Fig. S2E, F), which aligns with previous reports (17, 18). Under HFD conditions, *Mcu* BKO mice demonstrated glucose intolerance and insulin resistance (Fig. 2H, I), along with elevated levels of serum-free fatty acids (Fig. 2J). Notably, under HFD feeding, brown adipocytes in *Mcu* BKO mice showed decreased expressions of *Ucp1*, *Dio2*, and *Cpt1a* compared to those in wild-type mice (Fig. 2K). These results suggest that Mcu deficiency attenuates energy expenditure and lipid oxidation in brown adipocyte, leading to impaired insulin sensitivity and glucose tolerance.

### MCU is required for activation of thermogenesis against cold exposure

**Fig. 3:**
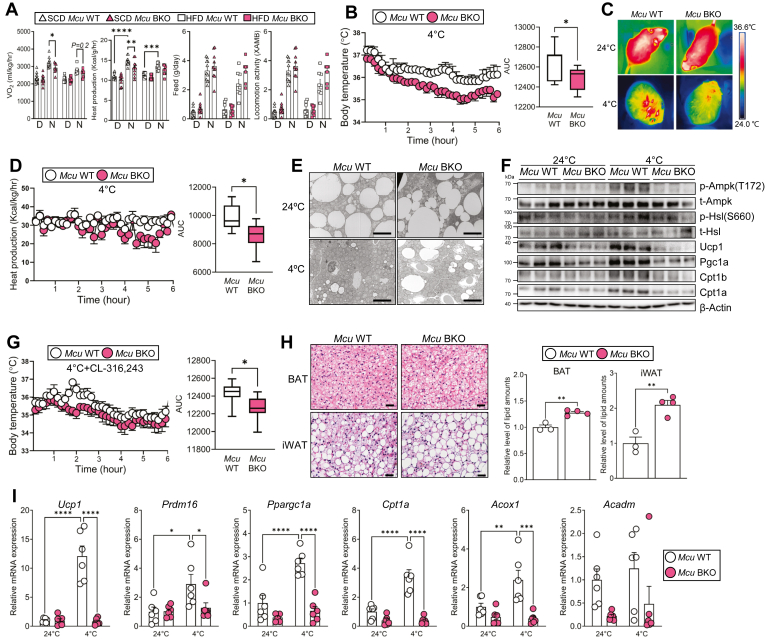
MCU is essential for thermogenic activation during cold exposure. A: Whole-body metabolic parameters in wild-type and *Mcu* BKO mice. “D” and “N” indicate light (day) and dark (night) phases, respectively. B: Core body temperature during 6-h cold exposure. C: Infrared thermal images after 6 h of cold exposure. D: Heat production during cold exposure. E: TEM images of BAT after cold exposure. Scale bar, 5 μm. F: Western blot analysis of mitochondrial proteins in BAT after cold exposure. G: Core body temperature in mice treated with CL-316,243 and cold exposure. H: Representative H&E staining (left) and lipid droplet quantification (right) of BAT. Scale bar, 40 μm. I: Thermogenic gene expression after 6-h cold exposure. Data represent mean ± SEM. Statistical significance was determined using unpaired two-tailed *t* test or one-way ANOVA. ∗*P* < 0.05; ∗∗*P* < 0.01; ∗∗∗*P* < 0.001; ∗∗∗∗*P* < 0.0001.

Given that *Mcu* BKO mice showed decreased expressions of genes related to energy expenditure, we hypothesized that Mcu deficiency in brown adipose tissue might affect heat production related to Ucp1 downregulation. As expected, oxygen consumption and heat production were decreased in *Mcu* BKO mice fed a SCD (Fig. 3A). We then exposed the mice to acute cold to activate nonshivering thermogenesis in BAT. Upon cold exposure, *Mcu* BKO mice exhibited lower core body temperature compared to wild type (Fig. 3B), and this difference persisted throughout the 24-h period (supplemental Fig. S3). In addition, the superficial body temperature, especially at the region of BAT, was also decreased in *Mcu* BKO mice (Fig. 3C), correlating with reduced heat production (Fig. 3D). Interestingly, while wild type mice showed decreased lipid droplet size in BAT due to cold-induced lipolysis, *Mcu* BKO mice exhibited significantly larger lipid droplets (Fig. 3E). BAT in *Mcu* BKO mice showed decreased expressions or inhibited activations of lipid-utilizing and heat-producing proteins including Hsl, Cpt1a, and Ucp1 (Fig. 3F). To rule out the possibility of altered sympathetic tone in *Mcu* BKO mice, we fully activated NST using a combination of cold exposure and β-adrenergic agonist CL-316,243 (19). Even under these conditions, we observed decreased core body temperature in *Mcu* BKO mice (Fig. 3G), along with diminished activation of brown adipose tissue and browning of inguinal white adipose tissue, evidenced by higher lipid contents (Fig. 3H). Gene expression profiling further corroborated these findings, showing reduced expressions of thermogenic genes, including *Ucp1* and *Cpt1a* (Fig. 3I). Taken together, these findings suggest that Mcu in BAT is crucial for the activation of thermogenesis in response to cold exposure.

### MCU is required for uncoupling and lipid utilization

**Fig. 4:**
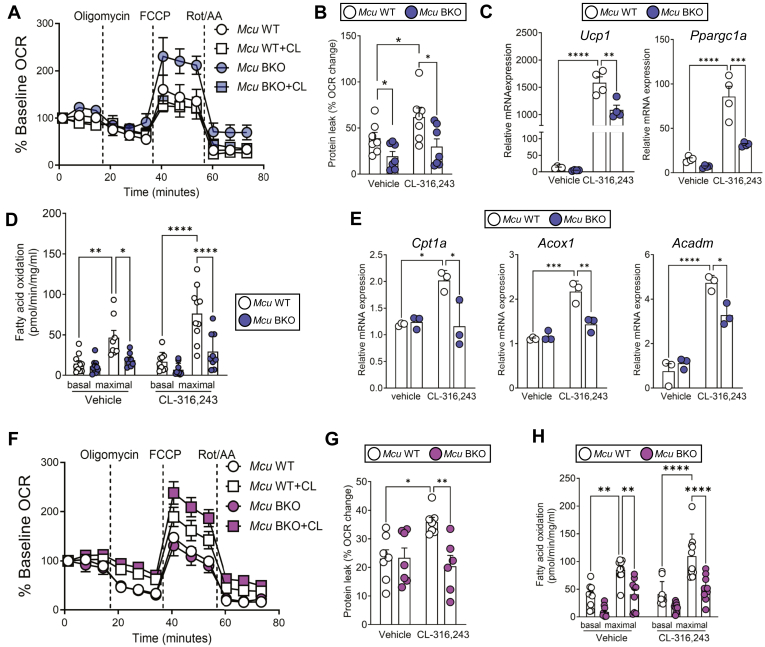
MCU is required for mitochondrial uncoupling and lipid utilization. A: Oxygen consumption rate (OCR) of primary brown adipocytes from wild-type and *Mcu* BKO mice after CL-316,243 treatment. B: Proton leak calculated from OCR traces shown in (A). C: Thermogenic gene expression in primary brown adipocytes. D: Etomoxir-sensitive OCR in primary brown adipocytes. E: Expression of β-oxidation–related genes in primary brown adipocytes. F: OCR in primary beige adipocytes from wild-type and *Mcu* BKO mice with CL-316,243. G: Proton leak calculated from OCR traces shown in (F). H: Etomoxir-sensitive OCR in beige adipocytes. Data represent mean ± SEM. Statistical significance was determined using one-way ANOVA. ∗*P* < 0.05; ∗∗*P* < 0.01; ∗∗∗*P* < 0.001; ∗∗∗∗*P* < 0.0001.

To explore the cell-autonomous effects of Mcu on the activation of thermogenic adipocytes, we assessed their cellular respiration. Upon activation by CL-316,243 treatment, primary brown adipocytes from *Mcu* BKO mice showed decreased proton leak, indicating a diminished uncoupling (20) (Fig. 4A, B and supplemental Fig. S4A). This reduction was accompanied by decreased gene expressions (Fig. 4C), consistent with observations in brown adipose tissue (Fig. 3I). We further confirmed this result in immortalized brown adipocytes with *Mcu* knockdown, which showed similar changes in thermogenic gene expression (supplemental Fig. S4B). Since β-adrenergic stimulation enhances mitochondrial fatty acid utilization (21), we examined β-oxidation in primary brown adipocytes of wild type and *Mcu* BKO mice. The brown adipocytes from *Mcu* BKO mice showed lowered rates of fatty acid oxidation (Fig. 4D), accompanied by reduced gene expression associated with β-oxidation (Fig. 4E). This reduction in β-oxidation-related genes was similarly observed in the siRNA knockdown model (supplemental Fig. S4C). To verify whether Mcu also influences beige adipocytes, we conducted primary cell cultures from inguinal white adipose tissue and induced their differentiation into beige adipocytes. Similarly, decreased proton leak and fatty acid utilization were also seen in beige adipocytes (Fig. 4F–H and supplemental Fig. S4D). Therefore, the reduced cellular respiration observed in thermogenic adipocytes lacking *Mcu* may be linked to lowered utilization of fatty acids and uncoupling activity for thermogenesis.

### MCU regulates mitonuclear signaling via ROS generation

**Fig. 5:**
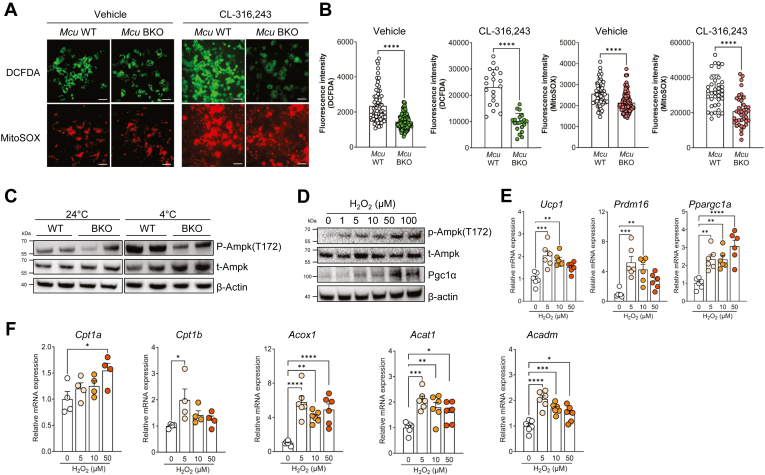
Mcu deficiency impairs ROS-dependent AMPK activation and thermogenic gene expression. A: DCFDA and MitoSOX staining of primary brown adipocytes from wild-type and *Mcu* BKO mice following CL-316,243 treatment. Scale bar, 25 μm. B: Quantification of ROS levels shown in (A). C: Western blot analysis of AMPK phosphorylation in BAT after cold exposure. D: Immunoblotting of AMPK phosphorylation and Pgc1α in brown adipocytes treated with H_2_O_2_. E, F: Expression of thermogenic and β-oxidation genes in wild-type primary brown adipocytes treated with H_2_O_2_. Data represent mean ± SEM. Statistical significance was determined using unpaired two-tailed *t* test or one-way ANOVA. ∗*P* < 0.05; ∗∗*P* < 0.01; ∗∗∗*P* < 0.001; ∗∗∗∗*P* < 0.0001.

**Fig. 6:**
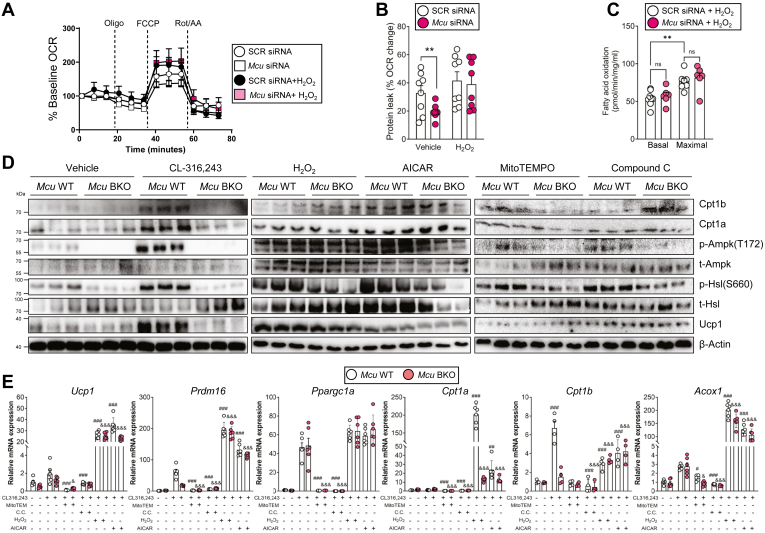
Exogenous H_2_O_2_ restores thermogenic signaling and gene expression in Mcu-deficient cells. A: OCR in scramble and siMcu-treated brown adipocytes with or without H_2_O_2_. B: Proton leak calculated from OCR traces shown in (A). C: FAO rate under H_2_O_2_ treatment. D: Western blot analysis of AMPK phosphorylation and β-oxidation gene expression under indicated treatments (CL-316,243, H_2_O_2_, AICAR, MitoTEMPO, Compound C). E: mRNA expression of thermogenic and FAO-related genes in treated brown adipocytes. Data represent mean ± SEM. Statistical significance was determined using unpaired two-tailed *t* test or one-way ANOVA. ∗*P* < 0.05; ∗∗*P* < 0.01; ∗∗∗*P* < 0.001; ∗∗∗∗*P* < 0.0001. #, versus CL-316,243-treated wild type; &, versus CL-316,243-treated *Mcu* BKO.

Since *Mcu* BKO mice not only altered mitochondrial respiration but also affected nuclear gene expressions, we speculated that the absence of Mcu might disrupt mitochondria-to-nuclear signaling required for lipid metabolism and thermogenesis in BAT. We focused on mtROS, as a mitonuclear signal (22, 23), and demonstrated that mtROS, as well as cytosolic ROS, were significantly lowered in brown adipocytes from *Mcu* BKO mice both in basal and CL-316,243-treated conditions (Fig. 5A, B). Concurrently, AMPK activation, typically observed in wild type mice upon cold exposure (24), was decreased in Mcu BKO mice (Fig. 5C). This led us to postulate the existence of a ROS-mediated mitonuclear signaling pathway that drives thermogenesis in adipose tissues. To validate this hypothesis, we examined the role of ROS in AMPK activation by directly applying H_2_O_2_ to primary brown adipocytes. Treatment with H_2_O_2_ dose-dependently increased AMPK phosphorylation in brown adipocytes from wild type mice (Fig. 5D), along with increased expression of genes related to thermogenesis and β-oxidation (Fig. 5E, F). To further support our hypothesis, we employed a rescue experiment. Exogenous H_2_O_2_ treatment abolished the reduction in proton leak caused by *Mcu* knockdown (Fig. 6A, B and supplemental Fig. S5A), indicating a restoration of thermogenic function. Furthermore, decreased fatty acid oxidation in *Mcu* deficiency was also restored by exogenous H_2_O_2_ treatment (Fig. 6C). Primary brown adipocytes from *Mcu* BKO mice maintained lower levels of AMPK phosphorylation under basal conditions or treating with CL-316,243. However, the appliinduces of H_2_O_2_ induce AMPK phosphorylation in *Mcu* BKO mice, similar to the response in wild-type mice (Fig. 6D). Importantly, genes related to thermogenesis and β-oxidation, which were reduced in *Mcu* knockout, were restored either by the induction of ROS with H_2_O_2_ or by treatment with AMPK activators (Fig. 6D, E and supplemental Fig. S5B, C). Conversely, when wild-type adipocytes were treated with a mitochondrial ROS scavenger Mito-TEMPO (25) or AMPK inhibitor compound C (26), AMPK phosphorylation diminished along with decreased expressions of genes which were upregulated by CL-316,243 treatment. There results demonstrate that mitochondria lacking Mcu leads to decreased mtROS production, subsequently impacting mitonuclear signaling for nuclear gene expression. Therefore, we concluded that, in the context of activated thermogenic adipocytes, Mcu not only acutely regulates thermogenic metabolism but also chronically modulates sustained thermogenesis through nuclear gene expression via ROS-AMPK pathway. ROS has been reported to activate Hif-1a, leading to changes in gene expression (27, 28). To determine whether Hif-1a is involved in mtROS-mediated mitonuclear signaling with AMPK, we examined gene expression in *Hif1a* knockdown cells in response to H_2_O_2_ (supplemental Fig. S5D). Even in the absence of Hif-1a, the target genes were upregulated by H_2_O_2_, suggesting the existence of Hif-1a-independent mitonuclear signaling induced by mtROS.

## Discussion

Brown or beige adipocytes are known to exert energy expenditure through various processes, including Ucp1-dependent uncoupling and creatine futile cycling at mitochondria, Ca^2+^ futile cycling at the endoplasmic reticulum, and triacylglycerol-fatty acid cycling (13). Since all these processes induce a systemic negative energy balance, the activation of thermogenic adipocytes has emerged as a potential therapeutic strategy against obesity, diabetes, and metabolic syndrome (29). Several approaches have been proposed to activate brown and beige adipocytes or to induce the browning of white adipocytes (30, 31). These approaches mainly target the induction of genes involved in lipid utilization, thermogenesis, and mitochondrial biogenesis. An additional strategy involves activating mitochondria, including enhanced beta-oxidation and citric acid cycle, which can increase ETC flow and electrochemical gradient for uncoupling (32). Mcu is known to activate mitochondria via activation of proteins such as pyruvate dehydrogenase, isocitrate dehydrogenase, α-ketoglutarate dehydrogenase, and complex III (33). In this study, we highlight the role of Mcu in thermogenic adipocytes concerning mitochondrial activation and mitonuclear signaling.

Mcu deletion resulted in impaired glucose tolerance and reduced insulin sensitivity, despite no significant difference in body weight. Consistently, knockdown of *Mcu* in brown adipocytes led to a marked decrease in glucose uptake (supplemental Fig. S2G). Given that glucose uptake by brown adipose tissue plays an important role in maintaining systemic glucose homeostasis and insulin sensitivity (34), it is plausible that systemic metabolic defects observed in *Mcu* BKO mice are at least partly attributable to reduced glucose utilization in BAT. Knockdown *of Mcu* in immortalized brown adipocytes resulted in decreased mitochondrial protein expression as well as reduced Ucp1, suggesting decreased energy expenditure in BAT. However, *Mcu* BKO mice exhibited body weight and energy expenditure comparable to the wild type under standard housing conditions. This muted phenotype may be explained by the fact that HFD induces whitening of BAT, leading to reduced mitochondrial activity and thermogenic capacity in both wild-type and *Mcu* BKO (35, 36). Under these conditions, the thermogenic defect caused by *Mcu* deletion may be masked. We therefore reasoned that the impact of *Mcu* deletion of BAT function would be more clearly revealed under conditions where BAT is robustly activated, such as during cold exposure or β3-adrenergic stimulation. *Mcu* BKO mice showed cold intolerance accompanied by decreased heat production, underscoring the importance of Mcu in NST. Because shivering thermogenesis could predominate during the initial hours of cold exposure, we extended the exposure duration to 24 h. Notably, *Mcu* BKO mice exhibited a sustained reduction in core body temperature, indicating that their cold intolerance results from a defect in NST. This is further supported by our findings of decreased oxygen consumption rates, diminished beta-oxidation activity, and related gene expressions as well as attenuated browning of WAT. Two previous studies using *Mcu* BKO models have reported conflicting results regarding cold exposure. One possible cause of this discrepancy is the differences in the mouse models used in these studies. The study that reported no difference in body temperature during cold exposure (37) used a model with exon 2 of the *Mcu* gene as the critical exon, while the study that reported cold intolerance (38) used exon 5, and our model used floxed mice targeting both exon 5 and exon 6. A recent reported a variant of *Mcu*, known as Mcu-SS, which does not include exons 1 to 4 (39). This variant lacks the mitochondrial targeting sequence, resulting in its function as a calcium channel in the plasma membrane. Applying this information, the model without cold intolerance likely expressed Mcu-SS, potentially enhancing cellular calcium influx and maintaining some level of thermogenesis, while the other models, including ours, likely suppressed the expression of all functional Mcu variants. Therefore, the expression of the remaining Mcu-SS could explain the different phenotypes observed in our model compared to the others. We found that *Mcu* removal led to decreased core body temperature and superficial skin temperature at the BAT area during cold exposure. It was due to decreased heat production and reductions in mitochondrial activity and lipid utilization at the cellular level. Therefore, we determined that Mcu in thermogenic adipocytes is essential for the process of increasing energy expenditure. This finding aligns with reports that kaempferol, known as an activator of Mcu (40), increases energy expenditure (41).

We found decreased *Ucp1* expression in *Mcu* knockout cells, which is accordance with the previous report demonstrated the role of Mcu on thermogenesis via complex formation with EMRE-UCP1 as a thermoporter (38). While alterations in the expressions of thermogenesis and beta-oxidation genes were noted upon deletion of *Mcu* in thermogenic tissues, the mechanism regulating nuclear gene expression by mitochondrial changes remains unclear. In this study, we propose Mcu-dependent generation of mtROS as a mitonuclear signal for activating thermogenic adipocytes. In mitochondria lacking Mcu, β -oxidation and citric acid cycle metabolism decreases, causing a restriction in electron transfer, evidenced from a decline in mitochondrial oxygen consumption. Consequently, mtROS, which is physiologically produced during electron transfer (42), also decreases. This decline in mtROS might be perceived in the nucleus as a state of mitochondrial inactivity, ultimately leading to diminished expressions of nuclear genes related to energy expenditure and thermogenesis. Thus, we suggest that mtROS serve not only as an indicator of mitochondrial activation but also as an initiator of mitochondrial adaptation to cold stress or beta-adrenergic stimulation in thermogenic adipocytes. Although ROS are considered as a pathogenic stressor disrupting metabolic homeostasis, they can act as a physiological signaling to have beneficial effects at very low concentrations, in the line with the hormesis theory (43). This signaling role of ROS is supported by previous studies demonstrating that low levels of H_2_O_2_ generated by glucose metabolism function as second messengers in pancreatic β-cells, regulating insulin secretion and redox adaptation (44, 45). Our findings are consistent with this model, as exogenous H_2_O_2_ restored AMPK activation and thermogenic gene expression in *Mcu*-deficient brown adipocytes. Our findings suggest that mtROS can activate AMPK through both indirect and direct mechanisms. Indirectly, mtROS impair mitochondrial ATP production, leading to a decreased ATP/ADP ratio, which in turn activates AMPK (46). Additionally, mtROS may directly oxidize specific cysteine residues on the AMPK α subunit, resulting in its activation, as suggested by other studies (47, 48). These dual mechanisms underscore the complex role of mtROS in regulating AMPK activity and, consequently, thermogenic gene expression. In addition to the AMPK-dependent effects, other lines of evidence suggest a broader role for mtROS in thermogenic regulation. For instance, increased energy expenditure in adipose tissue-specific *Sod2* (manganese superoxide dismutase) knockout mice (49), where ROS production is elevated, indicate that mtROS act as important regulators of thermogenesis. Although the thermogenic action of mtROS has been reported primarily occur through the modification of target proteins including Ucp1 (50), our research additionally reveals the presence of direct changes in gene expression. Therefore, in response to stress conditions requiring mitochondrial activation and thermogenesis, Mcu-dependent generation of mtROS could act as a mitonuclear signal, inducing long-term mitochondrial adaptation in thermogenic tissues.

There are several limitations in this study. First, we interpreted the decrease in body temperature of *Mcu* BKO mice under cold exposure because of reduced thermogenic adipocyte heat production, but we did not evaluate shivering thermogenesis, which is another aspect of body temperature maintenance. In brown adipocytes with impaired mitochondrial function due to *Mcu* knockout, there could be changes in the expression and secretion of BAT-derived “batokines” (51), which might affect body temperature regulation beyond nonshivering thermogenesis. Second, there is a discrepancy in phenotypes compared to previously reported *Mcu* floxed mice. In the model where exon 2 of *Mcu* was deleted, there was no change in body temperature under cold exposure (37), but in the models where exon 5 was deleted (38) and in our model (with exons 5 and 6 deleted), cold intolerance was observed. Given that splicing variants of *Mcu* have been reported (39), phenotypic differences might arise from changes in the cellular location or activity of Mcu proteins translated with critical exons removed; however, this was not addressed in our study. Third, we proposed mtROS as the signaling mechanism from mitochondria to the nucleus when Mcu is removed, but we lacked exploration of other candidates for mitonuclear signaling. Future studies are needed to analyze changes in the expression of mitochondria-derived peptides, such as MOTS-c (52), to further understand mitonuclear signaling changes due to mitochondrial calcium.

In summary, our findings establish Mcu as a pivotal regulator of thermogenic adipocyte function, orchestrating mitochondrial activation and mtROS-mediated mitonuclear signaling to drive energy expenditure and thermogenic gene expression. These insights provide a mechanistic framework for understanding adaptive thermogenesis and suggest that this pathway could serve as a tissue-specific target for improving energy expenditure in metabolic disease.

## Data availability

The data used in this study are available from the corresponding author upon reasonable request.

## Supplemental data

This article contains supplemental data.

## Conflict of interest

The authors declare that they have no conflicts of interest with the contents of this article.
