# Supplementary material for: Mitochondrial calcium uniporter is required for thermogenic adaptation mediated by reactive oxygen species signaling
Source: J Lipid Res. 2025 May 29;66(7):100834. doi: 10.1016/j.jlr.2025.100834 (PMC12256318; doi:10.1016/j.jlr.2025.100834)
Supplement: Supplementary Material [file mmc1.docx]

**Supplemental Figures**

**
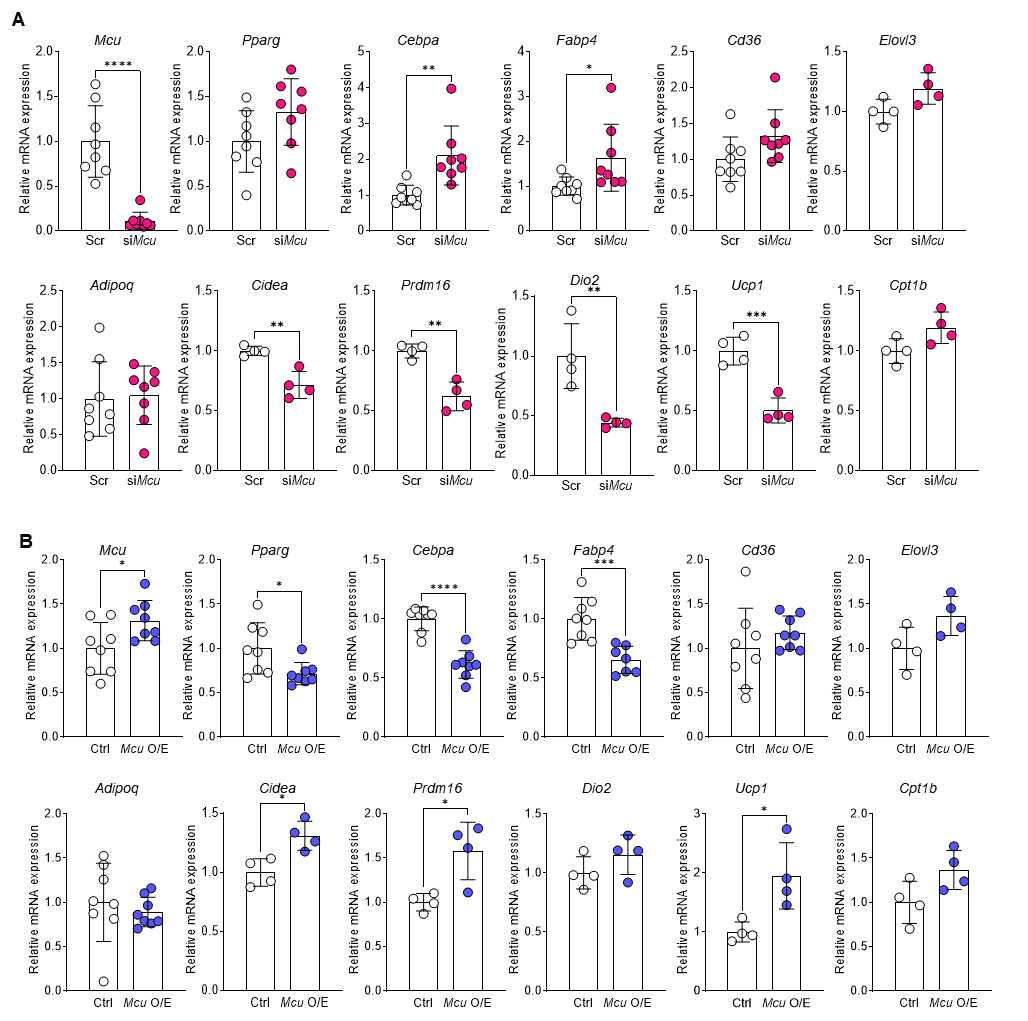
**

**Supplemental Fig. S1.** Expression of adipogenic and thermogenic genes upon *Mcu* knockdown or overexpression. (A) Relative mRNA expression of thermogenic and adipogenic markers in immortalized brown adipocytes transfected with *Mcu* siRNA. (B) Expression of the same markers in *Mcu*-overexpressing cells. Data represent mean ± SEM. Statistical significance was determined using unpaired two-tailed t test; *p < 0.05, **p < 0.01, ***p < 0.001, ****p < 0.0001.


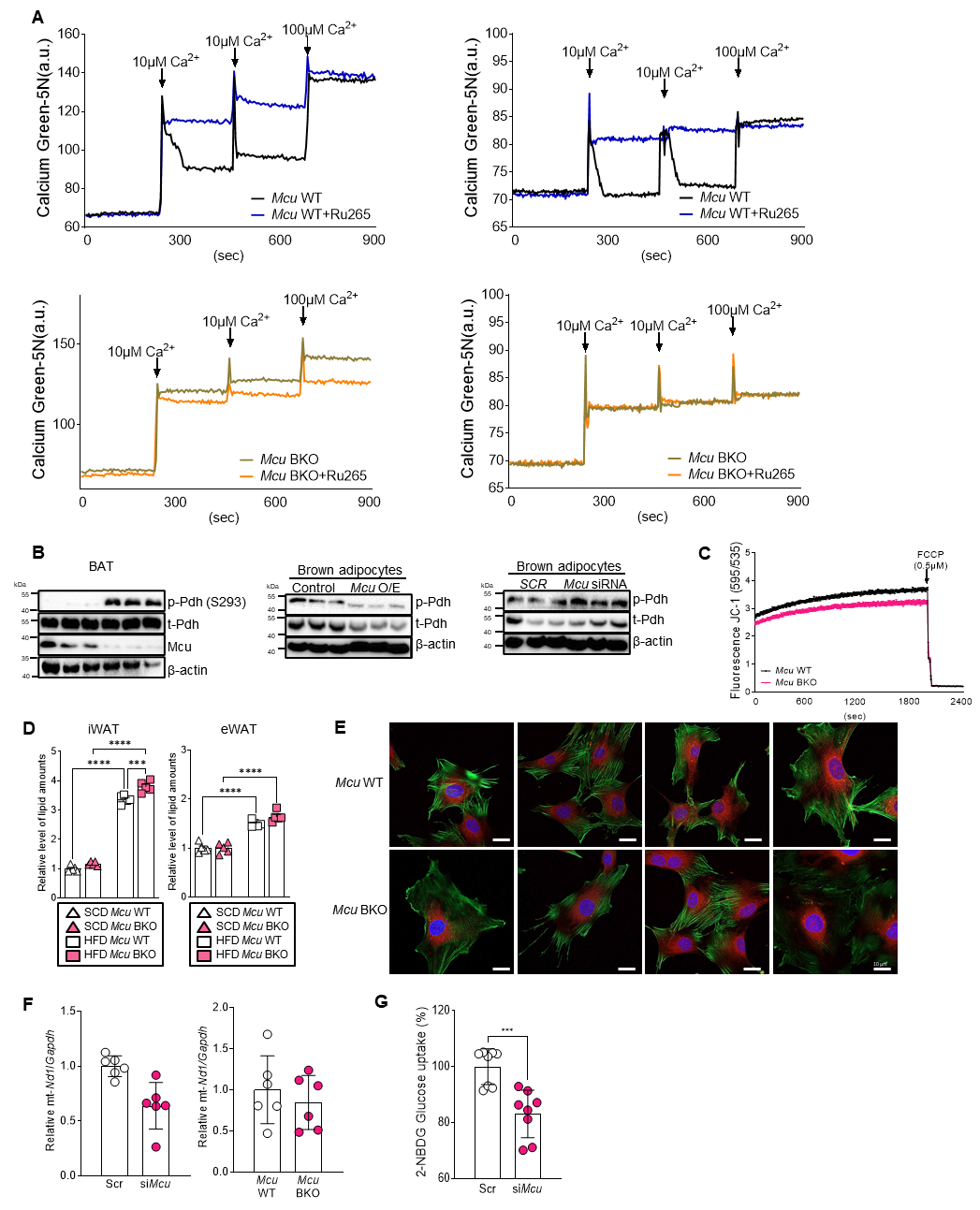


**Supplemental Fig. S2.** Mitochondrial characteristics and lipid accumulation in *Mcu*-deficient brown adipocytes. (A) Mitochondrial Ca^2+^ uptake assessed in *Mcu* WT and BKO adipocytes. (B) Western blot of phosphorylated PDH. (C) Mitochondrial membrane potential measured using JC-1 dye. (D) Quantification of lipid accumulation in iWAT and eWAT depots corresponding to the images shown in Figure 2E. (E) Fluorescence staining of primary brown adipocytes from *Mcu* BKO mice (blue: DAPI; red: MitoTracker; green: Alexa 488). Scale bar, 10 μm. (F) Mitochondrial DNA content (*mt-Nd1*) in *Mcu* knockdown cells and *Mcu* BKO tissues. (G) 2-NBDG uptake assay in primary brown adipocytes. Data represent mean ± SEM. Statistical significance was determined using unpaired two-tailed t test or one-way ANOVA where applicable; *p < 0.05, **p < 0.01, ***p < 0.001, ****p < 0.0001.

**Supplemental Fig. S3.** Time-course of core body temperature during cold exposure. Rectal temperature of *Mcu* WT and BKO mice was measured at the indicated time points during 24-hour cold exposure (4 °C) to assess core body temperature. Data represent mean ± SEM. Statistical significance was determined using unpaired two-tailed t test or one-way ANOVA where applicable; *p < 0.05, **p < 0.01, ***p < 0.001, ****p < 0.0001.


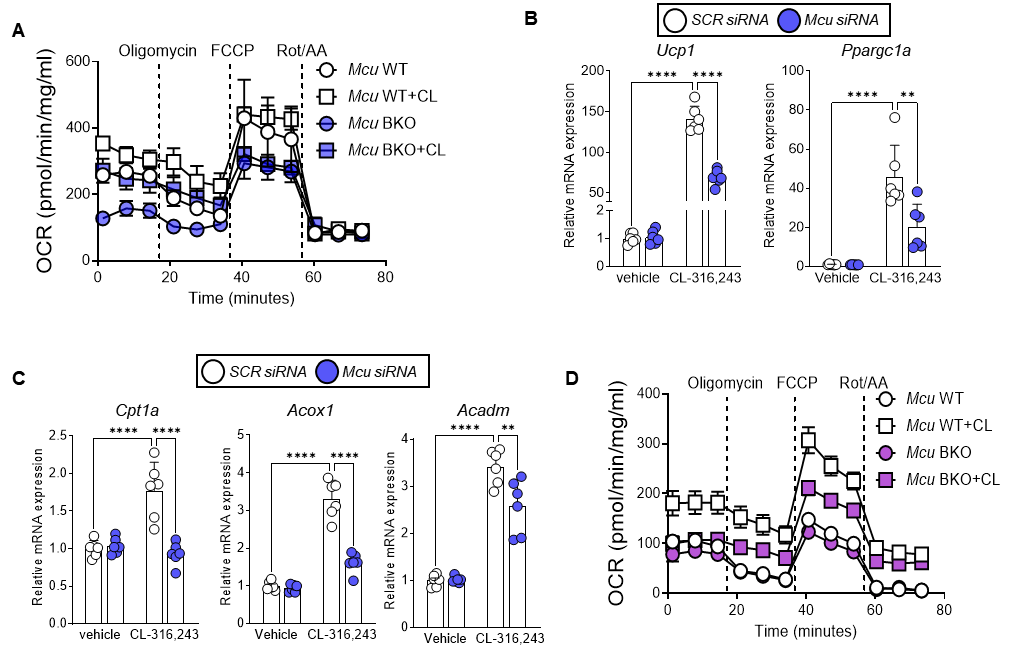


**Supplemental Fig. S4.** Mitochondrial respiration and gene expression in *Mcu*-deficient brown adipocytes. (A) Raw OCR traces corresponding to primary brown adipocytes shown in Fig. 4A. (B) Relative expression of thermogenic genes in immortalized brown adipocytes transfected with scramble or *Mcu* siRNA. (C) Relative expression of β-oxidation-related genes in immortalized brown adipocytes transfected with scramble or *Mcu* siRNA. (D) Raw OCR traces corresponding to primary beige adipocytes shown in Fig. 4F. Data represent mean ± SEM. Statistical significance was determined using unpaired one-way ANOVA; **p < 0.01, ****p < 0.0001.


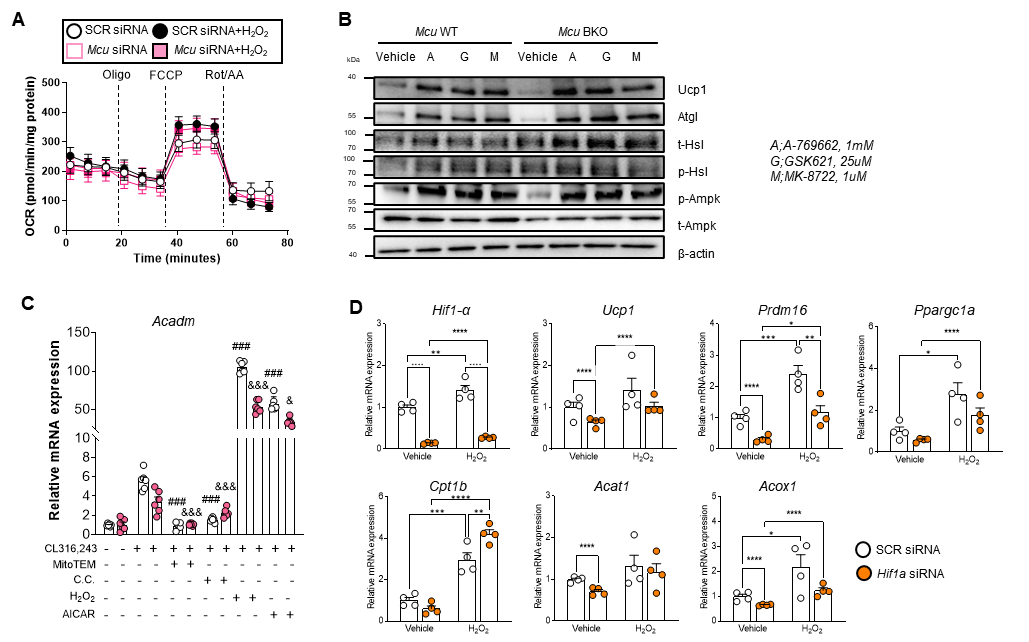


**Supplemental Fig. S5.** Pharmacological and genetic modulation of ROS/AMPK signaling in *Mcu*-deficient brown adipocytes. (A) Raw OCR traces of brown adipocytes shown in Figure 6A. (B) Western blot of phosphorylated AMPK and lipolytic proteins in *Mcu* WT and BKO cells treat with AMPK activators. (C) *Acadm* mRNA expression under AMPK modulator treatment. (D) Expression of Hif1a and thermogenic/β-oxidation genes in cells transfected with scramble or *Hif1a* siRNA and treated with vehicle or H_2_O_2_. Data represent mean ± SEM. Statistical significance was determined using one-way ANOVA; *p < 0.05, **p < 0.01, ***p < 0.001, ****p < 0.0001. #, vs CL-316,243-treated wild type; &, vs CL-316,243-treated *Mcu* BKO.

**Supplemental Tables**

**Table S1. Key resources table**

| REAGENT or RESOURCE | SOURCE | IDENTIFIER |
| --- | --- | --- |
| Antibodies | | |
| OXPHOS: Atf5a | Abcam | ab110413 |
| OXPHOS: Uqcrc2 | Abcam | ab110413 |
| OXPHOS: MtCo1 | Abcam | ab110413 |
| OXPHOS: Sdhb | Abcam | ab110413 |
| OXPHOS: Ndufb8 | Abcam | ab110413 |
| Mitochondrial calcium uniporter (Mcu) | Cell signaling | 14997S |
| Uncoupling protein 1 (Ucp1) | Cell signaling | 14670S |
| ATP synthase F1 subunit α (Atp5A) | Abcam | ab14748 |
| PPARG Coactivator 1 Alpha (Pgc1α) | Abcam | ab191838 |
| Carnitine Palmitoyltransferase 1A (Cpt1α) | Abcam | ab128568 |
| Carnitine Palmitoyltransferase 1B (Cpt1β) | Proteintech | 22170-1-AP |
| β-actin | Santa cruz | sc-69879 |
| Pyruvate Dehydrogenase E1 Component Subunit α (p-Pdh, Ser293) | Sigma | ABS204 |
| Pyruvate Dehydrogenase E1 Component Subunit α (t-Pdh) | Invitrogen | 459400 |
| 5' AMP-activated protein kinase (p-Ampk α (T183)) | Abcam | ab133448 |
| 5' AMP-activated protein kinase (p-Ampk α (T172)) | Cell signaling | 2535S |
| 5' AMP-activated protein kinase (t-Ampk α1+ α2) | Abcam | ab207442 |
| 5' AMP-activated protein kinase (t-Ampk α) | Cell signaling | 2532S |
| Hormone-sensitive lipase (p-Hsl, Ser565) | Cell signaling | 4137S |
| Hormone-sensitive lipase (p-Hsl, Ser660) | Cell signaling | 45804S |
| Hormone-sensitive lipase (t-Hsl) | Cell signaling | 4107S |
| Chemicals, peptides, and recombinant proteins | | |
| Dulbecco Modified Eagle Medium (DMEM) | Gibco | 11971-025 |
| Gibco Dulbecco's Modified Eagle Medium: Nutrient Mixture F12 (DMEM/F12) | Gibco | 11330-032 |
| Dexamethasone | Sigma | 102155389 |
| Indomethacin | Sigma | 17378-5G |
| 3-isobutyl-1-mrthylxanthine (IBMX) | Sigma | I7378 |
| Insulin | Sigma | 19278 |
| Rosiglitazone | Sigma | R2408 |
| 3,3',5-Triiodo-L-thyronine (T3) | Sigma | 101763263 |
| Fetal bovine serum (FBS) | Gibco | 16000-044 |
| Penicillin/Streptomycin | Gibco | 15140-122 |
| Trizol | Invitrogen | 15596018 |
| RIPA buffer | Invitrogen | 89901 |
| Protease inhibitor X100 | GenDEPOT | P3300-005 |
| Lipofectamine RNAiMAX | Invitrogen | 56531 |
| Lipofectamine 3000 | Invitrogen | 100022050 |
| Glucose | Agilent | 103577 |
| CL 316,243 | Sigma | C5976 |
| MitoTEMPO | Sigma | SML0737 |
| H_2_O_2_ (Hydrogen Peroxide) | Sigma | 216763 |
| AICAR | Sigma | A9978 |
| AMPK Inhibitor, Compound C | Sigma | 171260 |
| JC-1 | Invitrogen | T3168 |
| Calcium Green™-5N, Hexapotassium Salt, cell impermeant | Invitrogen | C3737 |
| Ru-265 | Sigma | SML2991 |
| Oligomycin | Sigma | 75351 |
| Carbonyl cyanide 4-(trifluoromethoxy)phenylhydrazone (FCCP) | Sigma | C2920 |
| Mitotracker | Invitrogen | M7512 |
| Alexa 488 | Invitrogen | A12379 |
| DAPI (4',6-diamidino-2-phenylindole, dihydrochloride) | Invitrogen | D1306 |
| Carboxy-DCFDA (5-(and-6)-Carboxy-2',7'-Dichlorofluorescein Diacetate), mixed isomers | Invitrogen | C369 |
| MitoSOX | Invitrogen | M36008 |
| Critical commercial assays | | |
| SuperScriptIII First-Strand | Invitrogen | Lot2520713 |
| Agilent Seahorse XF Substrate Oxidation Stress Test Kit | Agilent | 103015-100 |
| JC-1 Assay kit | Invitrogen | M34152 |
| Experimental models: Cell lines | | |
| Brown adipocyte | This paper | N/A |
| Primary brown adipocyte | This paper | N/A |
| Primary white adipocyte | This paper | N/A |
| Experimental models: Organisms/strains | | |
| Mouse: *Mcu* floxed | The Jackson Laboratory | 029817 |
| Mouse: *Ucp1* promoter-driven Cre-transgenic | The Jackson Laboratory | 024670 |
| Oligonucleotides | | |
| See Table S1 for primer sequences | This paper | N/A |
| Software and algorithms | | |
| ImageJ | NIH | https://imagej.net/ |
| GraphPad Prism 10 | GraphPad | RRID: SCR_002798 |
| Minispec Plus NF | Bruker | N/A |
| Image Lab | BIO-RAD | https://www.bio-rad.com/ |
| QuantStudio Real time software | Thermo Fisher | https://www.thermofisher.com/ |
| Other | | |
| Thermo imaging system | Teledyne FLIR | T530 |
| Comprehensive Lab Animal Monitoring System | Columbus Instrument | N/A |
| ELISA analyzer | Tecan | Infinite F-50 |
| High fat diet | Research diet | D12492 |
| Body temperature chip | STARR | G2 E-Mitter |
| Nuclear magnetic resonance spectroscopy (NMR) | Bruker | LF50 |
| Transmission Electron Microscope (TEM) | Hitachi | HT7800 |

**Table S2. Primer sequences used in this study**

| **Gene** | **Forward** | **Reverse** |
| --- | --- | --- |
| *Acat1* | CCCCATTGATTTTCCACTTG | AGCACAACCACACTGAATGC |
| *Acadm* | GGTTTGGCTTTTGGACAATG | TGACGTGTCCAATCTACCACA |
| *Acox1* | CACGGCTATTCTCACAGCAG | CAGGCTGTTAATGTCCACCA |
| *Adipoq* | CTCCACCCAAGGGAACTTGT | GGACCAAGAAGACCTGCATC |
| *Cd36* | GGCCAAGCTATTGCGACAT | CAGATCCGAACACAGCGTAGA |
| *Cidea* | ATCACAACTGGCCTGGTTACG | TACTACCCGGTGTCCATTTCT |
| *Cebpa* | TGCGCAAGAGCCGAGATAAA | TCACTGGTCAACTCCAGCAC |
| *Cpt1a* | AGCTCGCACATTACAAGGACA | CCAGCACAAAGTTGCAGGAC |
| *Cpt1b* | CAGCTTCCAAACGTCACTGC | ACAGAATCCAAGTACCGGTGAA |
| *Dio2* | TTGGGGTAGGGAATGTTGGC | TCCGTTTCCTCTTTCCGGTG |
| *Elovl3* | TCTTCCTGGCAATATTCAGTATCC | GCTTGAGGCCCACTGTAAAC |
| *Fabp4* | CACCGCAGACGACAGGAAG | GCACCTGCACCAGGGC |
| *Gapdh* | GAACTCCTCATGGGTCTGTAGTG | TGTTGTGGTACGTGCATAGCTG |
| *Hif1a* | TAAACACACAGCGGAGCTTT | CAGTGAAGCACCTTCCACG |
| *mt-ND1* | CCACGCTTCCGTTACGATCA | GTATGGTGGTACTCCCGCTG |
| *Pparg* | CCATTCTGGCCCACCAAC | AATGCGAGTGGTCTTCCATCA |
| *Ppargc1a* | GCCCAGGTACGACAGCTATG | CTTTGCCTCACTCAGGATTGG |
| *Prdm16* | CACGGTGAAGCCATTCATATGCG | CTCCGATGCTTGTTGAGGG |
| *Rplp0* | TCGTTGGAGTGACATCGTCT | TAGTTGGACTTCCAGGTCGC |
| *Ucp1* | CTTTGCCTCACTCAGGATTGG | ACTGCCACACCTCCAGTCATT |
